# A rare case report of gallbladder perforation caused by trauma

**DOI:** 10.3389/fmed.2025.1652501

**Published:** 2025-11-10

**Authors:** Keyu Chow, Yi He Wang, Zheng He

**Affiliations:** 1 Department of General Surgery, Shenzhen Bao'an Shiyan People's Hospital, Shenzhen, China; 2 Department of Urology, Shenzhen Bao'an Shiyan People's Hospital, Shenzhen, China

**Keywords:** gallbladder perforation, cholecystectomy, traumatic injury, surgery, case report

## Abstract

Isolated gallbladder perforation in blunt trauma is rare, accounting for approximately 2% of abdominal trauma laparotomy patients, often leads to delayed diagnosis due to non-specific symptoms. We report a 23-year-old male who sustained self-inflicted blunt trauma to the right upper abdomen while intoxicated and riding an electric bicycle, colliding with a bollard. He presented 11 h later with diffuse peritonitis, right upper quadrant pain, tachycardia (HR 109 bpm), and hypertension (BP 149/104 mmHg). Initial ultrasound showed 58 mm abdominal effusion and an atrophic gallbladder; paracentesis revealed bile. To identify the source of peritoneal effusion, rule out arterial extravasation, and assess other organ damage, contrast-enhanced computed tomography was performed—and this imaging confirmed gallbladder perforation. Laparoscopic cholecystectomy was performed, revealing a 2 cm perforation and 300 ml bile effusion. Post-operative course included antibiotics, nutrition support, and uneventful recovery with discharge on day 17. This case underscores the diagnostic pitfalls, as initial suspicion of liver injury was disproven by imaging. Rarity and non-specificity necessitate high suspicion in intoxicated patients, where alcohol may contribute to gallbladder vulnerability. Contrast-enhanced CT is crucial for confirmation, and cholecystectomy remains definitive treatment to prevent peritonitis. Clinicians should consider gallbladder perforation in blunt trauma, especially with alcohol involvement, to enable timely intervention and improve outcomes.

## Introduction

Gallbladder perforation secondary to traumatic injury is a rare condition. Reviewing the literature, among patients undergoing laparotomy for abdominal trauma, gallbladder perforation secondary to traumatic injury accounts for approximately 2% (1). Other rare causes of gallbladder perforation include emphysematous cholecystitis (2), post-chemotherapy and post-radiation (3), transarterial chemoembolization (4), gallbladder torsion (5), prematurity (6), biliary ascariasis (7), cholesterol crystal embolism (8), and type A aortic dissection (9). Most gallbladder injuries are complicated by cholecystitis (10). Typically, abdominal pain lacks obvious specificity, often requiring color Doppler ultrasound and contrast-enhanced computed tomography for diagnosis. Currently, cholecystectomy is recommended as the first-choice treatment (11).

This article adheres to the SCARE standard (12).

## Case report

A 23-year-old male worker, with a height of 1.57 m, weight of 42.5 kg, and a Body Mass Index (BMI) of 17.2, collided with a roadside bollard while riding an electric bicycle after consuming excessive alcohol, with the impact directly affecting his right upper abdomen, likely due to impaired coordination and muscle relaxation from intoxication. Immediately after the collision, he experienced right abdominal discomfort and pain, which gradually spread to the entire abdomen and intensified. He also had multiple episodes of nausea and vomiting with gastric contents. After self-monitoring at home, the symptoms showed no significant improvement, though they remained tolerable. Eleven hours after the injury, the abdominal pain worsened, prompting him to present to the emergency department of our hospital.

Given the patient's report of acute right-sided abdominal pain, the emergency department initially suspected a liver injury. The patient presented with the following vital signs: temperature (T): 36.5 °C; heart rate (HR): 109 beats per minute (bpm); respiratory rate (R): 20 breaths per minute (bpm); and blood pressure (BP): 149/104 mmHg. On physical examination, the patient had generalized abdominal rigidity, tenderness, and rebound tenderness, with tenderness being more prominent in the right upper quadrant. Following color Doppler ultrasound of the liver and gallbladder, only a depth of approximately 58 mm of abdominal effusion was detected. On color Doppler ultrasound, the liver was of normal size with homogeneous parenchyma, and hepatic ductal structures were well-visualized. The gallbladder appeared contracted. To clarify the cause of the traumatic abdominal pain and guide subsequent treatment, the patient was admitted to the Department of General Surgery.

Upon admission to the Department of General Surgery, the patient denied a history of hepatobiliary diseases as well as hypertension, and reported no previous surgical history. To determine the nature of the abdominal effusion as early as possible, abdominal paracentesis was performed under bedside ultrasound guidance immediately after admission, and dark yellow fluid was aspirated; this fluid was initially considered as bile. Furthermore, the nature of the fluid obtained via paracentesis facilitates targeted assessment of the injury status of abdominal organs during the subsequent review of abdominal contrast-enhanced CT images. Liver function tests showed significantly elevated levels: total bilirubin (39.40 μmol/L), direct bilirubin (18.60 μmol/L), indirect bilirubin (20.80 μmol/L), γ-glutamyl transferase (γ-GT, 187.00 U/L), alanine aminotransferase (ALT, 136.00 U/L), and aspartate aminotransferase (AST, 299.00 U/L).

**Figure 1:**
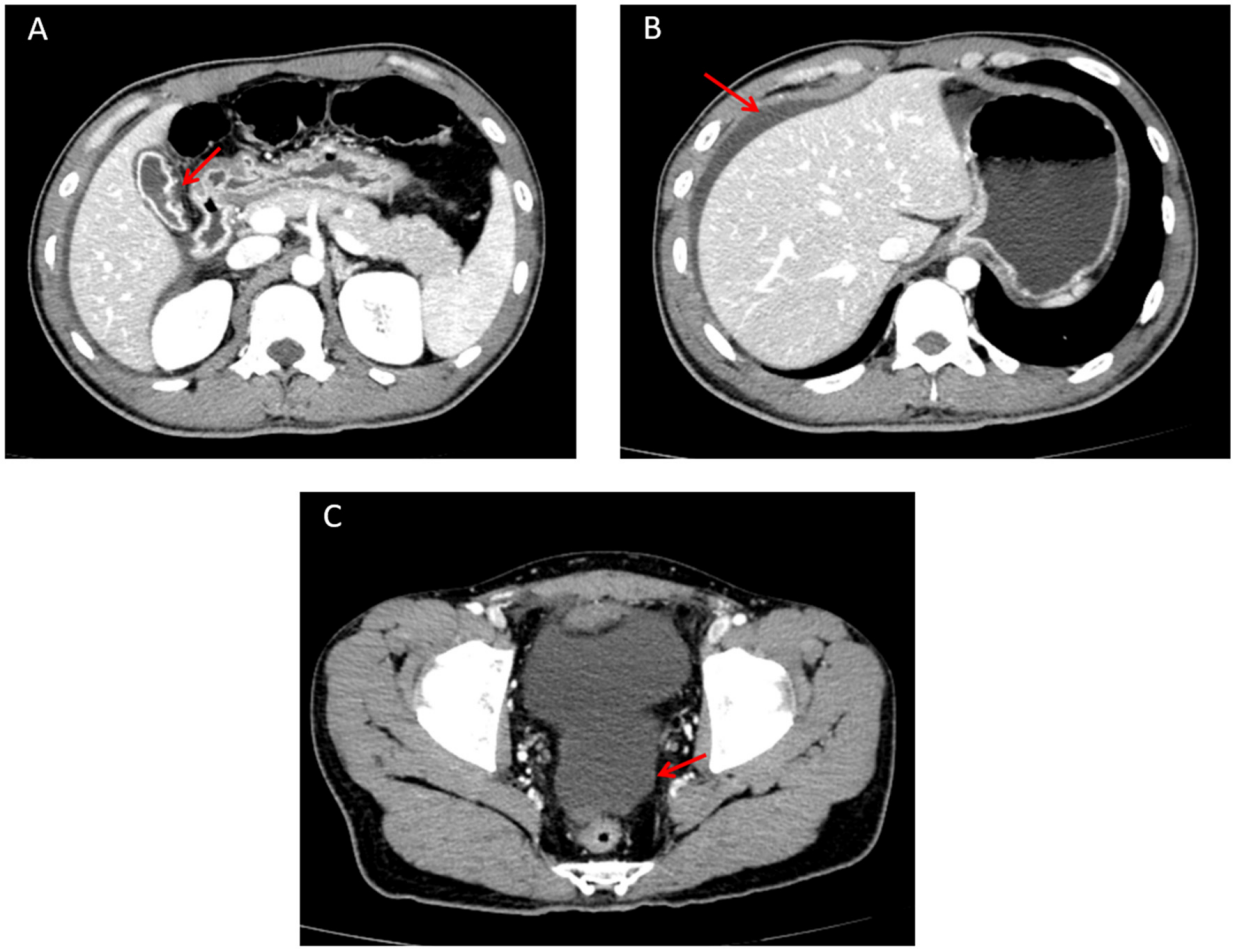
Abdominal contrast-enhanced CT image. **(A)** Perforated gallbladder (showing interrupted continuity and indistinct margins of the gallbladder wall adjacent to the duodenum) on abdominal computed tomography. **(B)** Perihepatic fluid collection, with the liver demonstrating a smooth outer margin, normal morphology, size, and hepatic lobe proportions, on abdominal computed tomography. **(C)** Pelvic fluid collection on abdominal computed tomography.

Concurrently, an urgent contrast-enhanced abdominal CT scan was performed, and the radiology department issued a critical value report as follows: the gallbladder is not enlarged but has a thickened wall with interrupted continuity and indistinct margins adjacent to the duodenum; no gallstones are seen in the lumen (Figure 1A). The liver has a smooth outer margin, normal morphology, size, and hepatic lobe proportions. No intrahepatic or extrahepatic bile duct dilatation is observed, and the hepatic hilum shows a clear structure without abnormal density (Figure 1B). Additionally, a pelvic fluid collection is noted on abdominal computed tomography (Figure 1C). After fully informing the patient and family, surgical exploration was immediately initiated.

**Figure 2:**
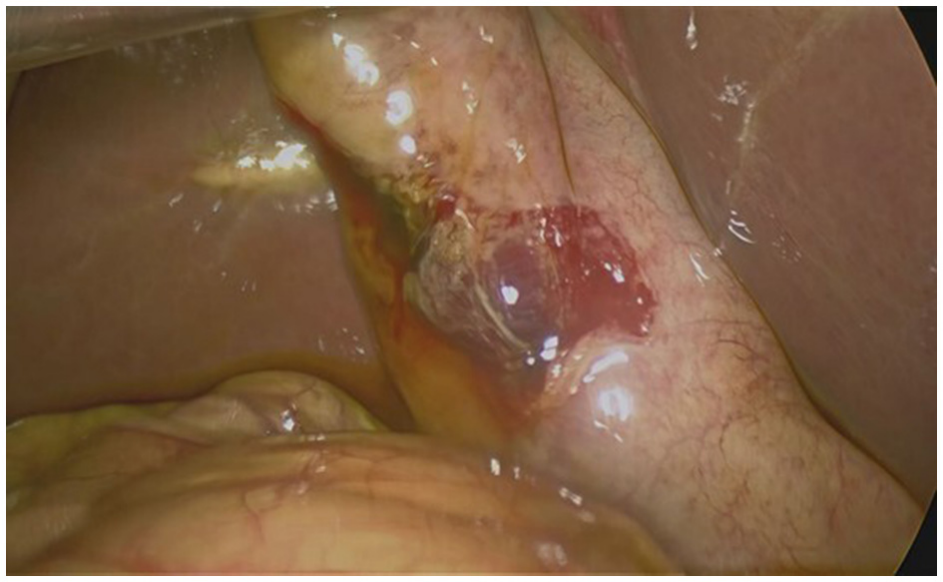
Laparoscopic view of the ruptured gallbladder.

**Figure 3:**
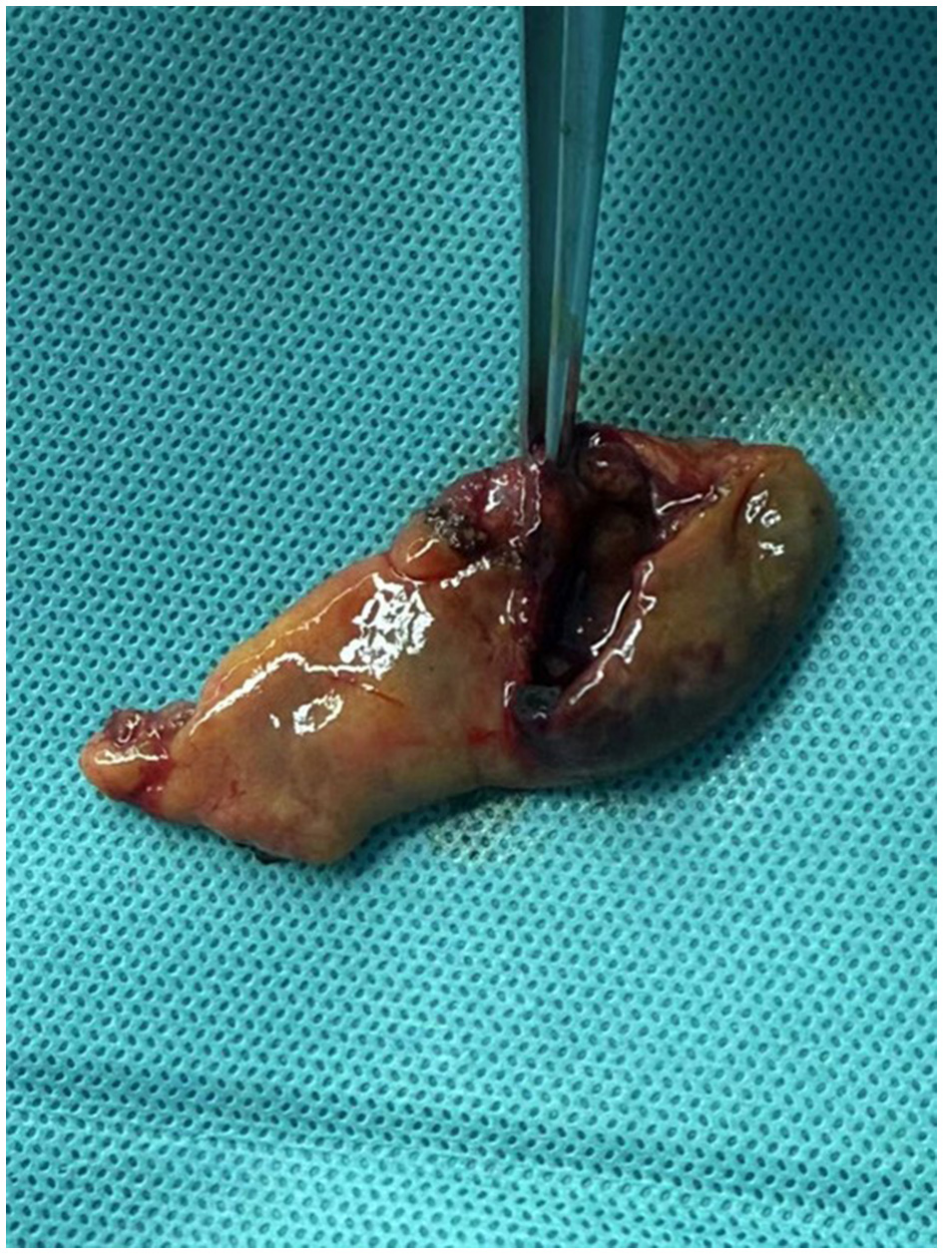
Gross specimen of the ruptured gallbladder.

During laparoscopic exploration, approximately 300 ml of bile was found in the abdominal cavity. The gallbladder was atrophic (5 cm × 4 cm; Figure 2), with a 2 cm rupture site showing necrotic tissue and congestion at the edge (Figure 3). A small area of liver contusion was noted near the gallbladder bed, while the diaphragmatic surface of the liver was smooth. Exploration of the stomach, duodenum, jejunum, ileum, colon, rectum, pancreas, and kidneys revealed no abnormalities. Thus, laparoscopic cholecystectomy for gallbladder perforation was performed under general anesthesia, with successful outcomes.

Post-operatively, the patient received antibiotics, fluid resuscitation, acid suppression, and analgesia. The pathological report showed focal gallbladder wall rupture with inflammatory necrotic tissue and extensive coagulation. To rule out the occurrence of delayed post-operative organ injury and intraoperative missed injuries, a follow-up abdominal CT scan was performed, which confirmed no pathological abnormalities (13). Given the patient's compromised early post-operative nutritional status, targeted and adequate nutritional support was promptly initiated. Following nutritional optimization, the patient achieved uneventful recovery and was discharged on post-operative day 17, with satisfaction expressed toward the entire clinical management and care.

## Discussion

In this rare case, the patient presented with right-sided upper abdominal pain as the initial symptom. Given the higher prevalence of liver injury in traumatic abdominal trauma, liver injury was initially suspected. Abdominal paracentesis yielded bile. However, normal liver findings, signs of abnormal peritoneal effusion, and gallbladder atrophy on ultrasound contradicted this hypothesis. Although liver injury was initially suspected due to its high prevalence in abdominal trauma, this hypothesis was contradicted by the ultrasound findings of a normal size liver, abnormal peritoneal effusion, and gallbladder atrophy, in conjunction with bile-positive abdominal aspirate. As peritonitis worsened, the patient developed elevated blood pressure, and we immediately performed bedside ultrasound-guided paracentesis. We then considered rare causes of gallbladder and biliary rupture. Bile obtained via paracentesis supported our suspicion, though concurrent organ rupture could not be ruled out.

Color Doppler ultrasound serves as a valuable initial diagnostic modality for abdominal trauma. For emergency physicians, this modality primarily facilitates the detection of intra-abdominal fluid and offers inherent advantages, including convenience, safety, high diagnostic accuracy for parenchymal organ injuries and peritoneal effusion, a significantly shorter overall diagnostic workflow compared with computed tomography, no need for patient transfer, no radiation exposure, and low cost (14, 15). In our hospital, the emergency department specifically is equipped with bedside color Doppler ultrasound devices to support this purpose. In the present case, color Doppler ultrasound proved valuable for the rapid identification of peritoneal effusion and an atrophic gallbladder. The subsequent analysis of peritoneal aspirate, which revealed bile, further supported the diagnosis of gallbladder rupture. However, the utility of ultrasound was limited in definitively excluding injuries to other solid organs (16). Thus, further evaluation with contrast-enhanced abdominal CT is clinically indicated. Ultimately, contrast-enhanced abdominal CT confirmed the diagnosis of traumatic gallbladder perforation. This further confirms that contrast-enhanced abdominal CT is the gold standard for diagnosing traumatic conditions (14).

Previous literature indicates that isolated gallbladder injury presents with non-specific early symptoms, making initial diagnosis challenging due to limited case reports (1). This case shows similarities to other isolated blunt trauma case reported by Pham et al. (17), where patients experienced delayed diagnosis primarily due to non-specific symptoms and initial suspicion of liver injury. In this case, right upper abdominal pain initially prompted suspicion of liver injury rather than gallbladder injury. Diffuse peritonitis from bile leakage further complicated diagnosis. The atrophic gallbladder prevented accurate visualization on ultrasound, highlighting that contrast-enhanced abdominal CT is essential when ultrasound is non-diagnostic (18). Endoscopic retrograde cholangiopancreatography (ERCP) may aid diagnosis when differentiating between liver and gallbladder injury in cases of massive bile leakage for a temporary biliary stent placement for conservative treatment (17, 19). However, in our case, contrast-enhanced CT enabled definitive diagnosis, rendering ERCP unnecessary. Laboratory abnormalities in transaminase and bilirubin levels can also guide diagnosis. Elevated transaminases should alert clinicians to potential hepatobiliary injury during trauma (11).

A review of the literature indicates that the primary cause of gallbladder rupture is the distended state of the gallbladder that suffers blunt trauma. For instance, the gallbladder, which is full of bile and in a distended state during the morning fasting period, is also susceptible to perforation when subjected to blunt trauma (20). In our case, alcohol might have contributed to the gallbladder perforation, as it induced muscle relaxation and gallbladder distension, and these effects caused the gallbladder to be potentially vulnerable to direct trauma. Similarly, analogous cases have been documented in which gallbladder rupture occurred in patients who were involved in traffic accidents after alcohol consumption (17, 19). Alcohol ingestion stimulates gastrin secretion, increasing bile production and gallbladder distension. Additionally, alcohol induces spasm of the Sphincter of Oddi, predisposing the gallbladder to rupture during trauma (21). Excessive alcohol consumption also relaxes abdominal muscles, further increasing the risk of gallbladder perforation after trauma (22).

Regarding treatment, while some teams use absorbable barbed sutures for laparoscopic gallbladder repair (23), and others attempt conservative management, which however ends in failure and requires delayed surgery (19). No unified guideline exists for isolated blunt gallbladder injury. According to the modified Niemeier classification system for gallbladder perforation referenced in the study by Aydogdu (24), this classification comprises three types, and our case falls into Type I. Specifically, Type I is defined as acute free perforation complicated with biliary peritonitis, Type II as subacute perforation associated with pericholecystic abscess, and Type III as chronic perforation accompanied by cholecystoenteric fistula. Aydogdu et al. (24) proposed that laparoscopic cholecystectomy should be the first-line treatment for modified Niemeier Type I gallbladder perforation, as this surgical approach not only reduces the incidence of complications but also shortens the hospital stay. Meanwhile, for patients with a pre-operatively confirmed diagnosis of gallbladder perforation, laparoscopic surgery should be prioritized as the initial surgical approach. Aydogdu et al. (24) argues that laparoscopy should be the initial surgical approach for patients pre-diagnosed with gallbladder perforation (24). In this case, paracentesis and enhanced abdominal CT enabled timely diagnosis, and laparoscopic exploration followed by cholecystectomy prevented deterioration from bile peritonitis and abdominal infection. The patient was discharged smoothly after surgery.

## Conclusion

Isolated blunt gallbladder injury is rare and clinical presentation is often non-specific. Therefore, emergency physicians should maintain a high index of suspicion for this injury, particularly in trauma patients with a history of alcohol consumption, where it may exacerbate gallbladder vulnerability through distension and muscle relaxation. While color Doppler ultrasound and diagnostic peritoneal aspiration can provide supportive evidence, contrast-enhanced abdominal computed tomography remains the cornerstone for definitive diagnosis to rule out concurrent injuries. Laparoscopic cholecystectomy is the treatment of choice and is associated with favorable outcomes. Early recognition and intervention can significantly improve prognosis in these cases.

## Data Availability

The raw data supporting the conclusions of this article will be made available by the authors, without undue reservation.

## References

[1] Kwan BYM Plantinga P Ross I. Isolated traumatic rupture of the gallbladder. Radiol Case Rep. (2015) 10:1029. doi: 10.2484/rcr.v10i1.1029 27408658 PMC4921154

[2] Misir AP Vahora I Unbehaun G Patel C Tiesenga F. Perforated emphysematous cholecystitis: a race against time. Cureus. (2023) 15:e35123. doi: 10.7759/cureus.35123 36945264 PMC10024972

[3] Zhang J Shen G Shi Y Zhang C Hong D Jin L . Spontaneous acalculous gallbladder perforation in a man secondary to chemotherapy and radiation: a rare case report. Medicine. (2018) 97:e0674. doi: 10.1097/MD.0000000000010674 29742709 PMC5959403

[4] Son MY Han BH Lee SU Yun BC Seo KI Huh JD. Gallbladder perforation after transarterial chemoembolization in a patient with a huge hepatocellular carcinoma. Korean J Gastroenterol. (2020) 75:351–5. doi: 10.4166/kjg.2020.75.6.351 32581207 PMC12285765

[5] Wood BE Trautman J Smith N Putnis S. Rare case report of acalculous cholecystitis: gallbladder torsion resulting in rupture. SAGE Open Med Case Rep. (2019) 7:2050313x18823385. doi: 10.1177/2050313X18823385 30719303 PMC6349984

[6] Lu YY Lai HS Hsieh WS Hsu WM. Ischemic gallbladder perforation in a premature infant. J Pediatr Surg. (2008) 43:E31–2. doi: 10.1016/j.jpedsurg.2008.02.070 18558162

[7] Sharma A Jariwala P Kaur N. Biliary ascariasis presenting with gangrenous perforation of the gall bladder: report of a case and brief review of literature. Trop Doct. (2018) 48:242–5. doi: 10.1177/0049475518768103 29649951

[8] Tappenden J Suvarna SK Ackroyd R Shrestha BM. Cholesterol crystal embolism leading to perforation of the gallbladder. Hepatobiliary Pancreat Dis Int. (2007) 6:653–5. doi: 10.1136/gut.2007.126417corr1 18086636

[9] Jha NK Kumar RA Ayman M Khan JA Cristaldi M Ahene C . Ischemic gall bladder perforation: a complication of type A aortic dissection. Ann Thorac Surg. (2013) 95:e155–6. doi: 10.1016/j.athoracsur.2012.11.055 23706468

[10] Hosaka A Nagayoshi M Sugizaki K Masaki Y. Gallbladder perforation associated with carcinoma of the duodenal papilla: a case report. World J Surg Oncol. (2010) 8:41. doi: 10.1186/1477-7819-8-41 20487525 PMC2887867

[11] Jaggard MK Johal NS Choudhry M. Blunt abdominal trauma resulting in gallbladder injury: a review with emphasis on pediatrics. J Trauma. (2011) 70:1005–10. doi: 10.1097/TA.0b013e3181fcfa17 21610404

[12] Sohrabi C Mathew G Maria N Kerwan A Franchi T Agha RA. The SCARE 2023 guideline: updating consensus Surgical CAse REport (SCARE) guidelines. Int J Surg. (2023) 109:1136–40. doi: 10.1097/JS9.0000000000000373 37013953 PMC10389401

[13] Elbanna KY Mohammed MF Huang SC Mak D Dawe JP Joos E . Delayed manifestations of abdominal trauma: follow-up abdominopelvic CT in posttraumatic patients. Abdom Radiol. (2018) 43:1642–55. doi: 10.1007/s00261-017-1364-4 29051983

[14] Ghafouri HB Zare M Bazrafshan A Modirian E Farahmand S Abazarian N. Diagnostic accuracy of emergency-performed focused assessment with sonography for trauma (FAST) in blunt abdominal trauma. Electron Physician. (2016) 8:2950–3. doi: 10.19082/2950 27790349 PMC5074755

[15] He NX Yu JH Zhao WY Gu CF Yin YF Pan X . Clinical value of bedside abdominal sonography performed by certified sonographer in emergency evaluation of blunt abdominal trauma. Chin J Traumatol. (2020) 23:280–3. doi: 10.1016/j.cjtee.2020.07.001 32762981 PMC7567895

[16] Radwan MM Abu-Zidan FM. Focussed Assessment Sonograph Trauma (FAST) and CT scan in blunt abdominal trauma: surgeon's perspective. Afr Health Sci. (2006) 6:187–90. 17140344 10.5555/afhs.2006.6.3.187 PMC1831890

[17] Pham HD Nguyen TC Huynh QH. Diagnostic imaging in a patient with an isolated blunt traumatic gallbladder injury. Radiol Case Rep. (2021) 16:2557–63. doi: 10.1016/j.radcr.2021.06.036 34306287 PMC8283152

[18] Birn J Jung M Dearing M. Isolated gallbladder injury in a case of blunt abdominal trauma. J Radiol Case Rep. (2012) 6:25–30. doi: 10.3941/jrcr.v6i4.941 22690293 PMC3370695

[19] Egawa N Ueda J Hiraki M Ide T Inoue S Sakamoto Y . Traumatic gallbladder rupture treated by laparoscopic cholecystectomy. Case Rep Gastroenterol. (2016) 10:212–7. doi: 10.1159/000437046 27462188 PMC4924469

[20] Lu PH Tran NH Van Nguyen C Tran Viet H. A blunt gallbladder trauma: a rare and easily overlooked case report. J Surg Case Rep. (2025) 2025:rjaf276. doi: 10.1093/jscr/rjaf276 40337543 PMC12056503

[21] Shah A Cho T Bokhari F. Isolated traumatic gallbladder injury: a rare case. Cureus. (2023) 15:e43982. doi: 10.7759/cureus.43982 37746348 PMC10516145

[22] Schachter P Czerniak A Shemesh E Avigad I Lotan G Wolfstein I. Isolated gallbladder rupture due to blunt abdominal trauma. HPB Surg. (1989) 1:359–62. doi: 10.1155/1989/95937 2487076 PMC2423544

[23] Liu DL Pan JY Huang TC Li CZ Feng WD Wang GX. Isolated traumatic gallbladder injury: a case report. World J Gastrointest Surg. (2023) 15:2639–45. doi: 10.4240/wjgs.v15.i11.2639 38111759 PMC10725536

[24] Aydogdu YF Gülçek E Koyuncuoglu AC Büyükkasap Ç Dikmen K. Minimally invasive approach in a rare emergency surgery, gallbladder perforation. BMC Surg. (2024) 24:207. doi: 10.1186/s12893-024-02495-z 38987756 PMC11234621

